# Supplementary material for: Complexing the Marine Sesquiterpene Euplotin C by Means of Cyclodextrin-Based Nanosponges: A Preliminary Investigation
Source: Mar Drugs. 2022 Oct 29;20(11):682. doi: 10.3390/md20110682 (PMC9692710; doi:10.3390/md20110682)
Supplement: Supplementary file 1 [file marinedrugs-20-00682-s001.zip › marinedrugs-1935814-supplementary.pdf]

Supplementary Material

Complexing the Marine Sesquiterpene Euplotin C by Means of Cyclodextrin-based Nanoponges: A Preliminary Investigation

Alessandra Bertoli, Anthea LoBue, Luca Quattrini, Stefania Sartini, Beatrice Polini, Sara Carpi, Francesco Paolo Frontini, Graziano Di Giuseppe, Graziano Guella, Paola Nieri, and Concettina La Motta.

Table of Content

**Figure S1**	ATR-FTIR spectrum of euplotin C.
**Figure S2**	ATR-FTIR spectrum of β-cyclodextrin-based nanosponges.
**Figure S3**	ATR-FTIR spectrum of the loaded EC-NS complex.
**Figure S4**	ATR-FTIR spectrum of the 1:1 physical mixture of euplotin C and β-cyclodextrin-nanosponges.
**Figure S5**	Superimposition of the ATR-FTIR spectra of euplotin C (red), the loaded EC-NS complex (green) and the binary 1:1 mixture of EC and NS (black).
**Figure S6**	^{1}H-NMR spectrum of β-cyclodextrin-based nanosponges, recorded in D_2O.
**Figure S7**	Graphical representation of the workflow putted in place to recover pure euplotin C from *E. crassus* SSt22 strain culture.
**Figure S8**	RP-C18-TLC analysis of samples A-F obtained by SPE fractionation of *E. crassus* cell pellets extraction.
**Figure S9**	Semi-preparative HPLC analysis of euplotin C fractions obtained by SPE purification.
**Figure S10**	^{1}H-NMR spectrum of euplotin C, recorded in $CDCl_3$-d_1.
**Figure S11**	^{13}C-NMR spectrum of euplotin C, recorded in $CDCl_3$-d_1.
**Figure S12**	LC-PDA-ESI-MS quali-quantitative analysis of euplotin C.
**Table S1**	Parent/adduct and daughter ions obtained by direct infusion ESI-MS analyses of unloaded β-cyclodextrin-based nanosponges (NS), monomeric β-cyclodextrin, and euplotin C extracted from loaded β-cyclodextrin-based nanosponges by means of either apolar (EC-NS1) or polar (EC-NS2) solvents.

Figure S1. ATR-FTIR spectrum of euplotin C.

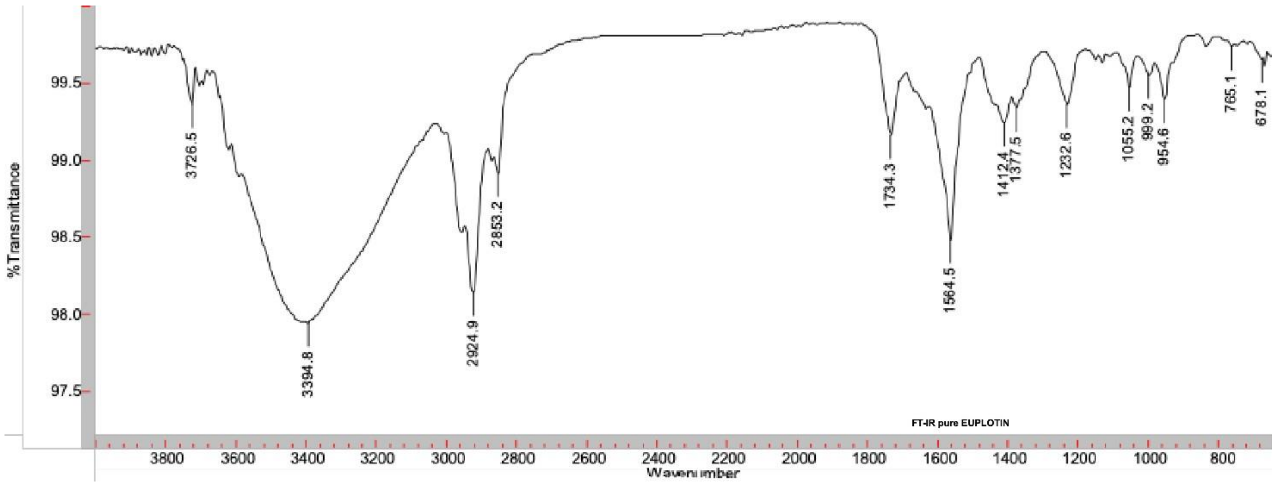

Figure S2. ATR-FTIR spectrum of β-cyclodextrin-based nanosponges.

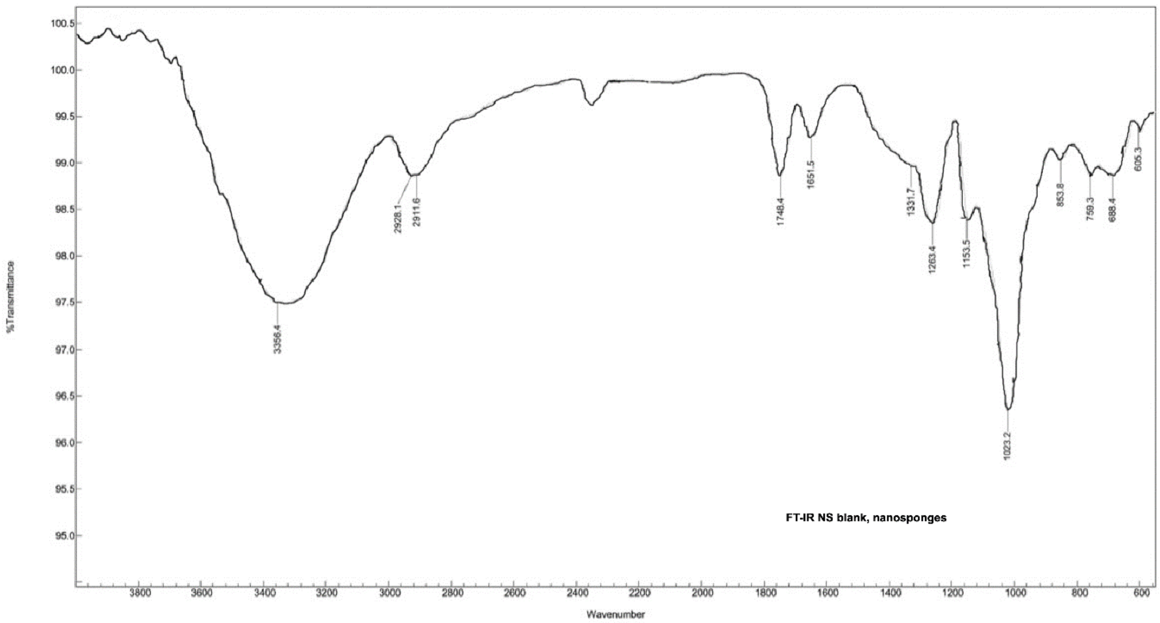

Figure S3. ATR-FTIR spectrum of the loaded EC-NS complex.

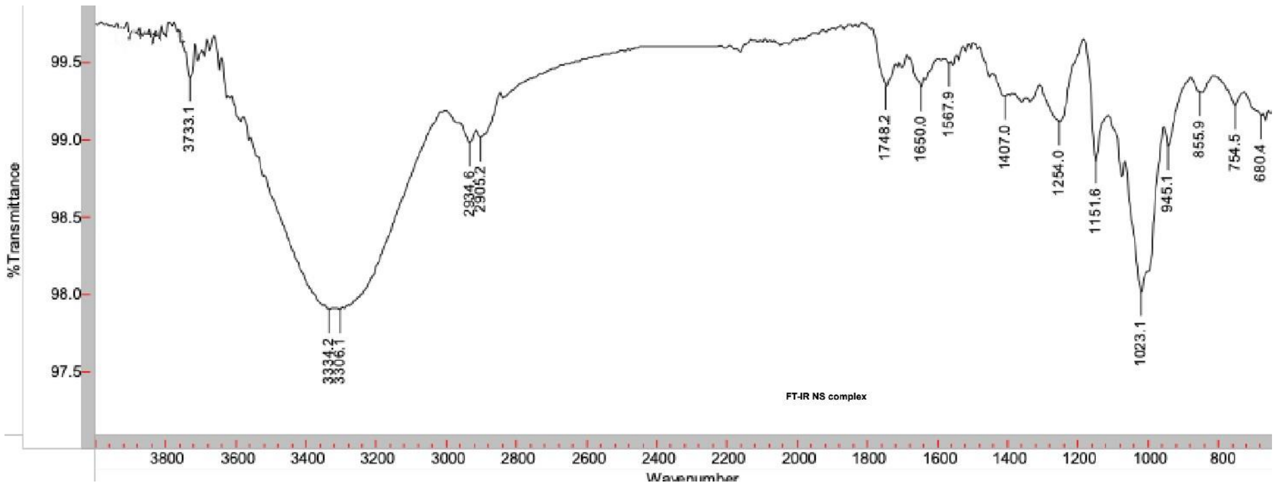

Figure S4. ATR-FTIR spectrum of the 1:1 physical mixture of euplotin C and β-cyclodextrin-based nanosponges.

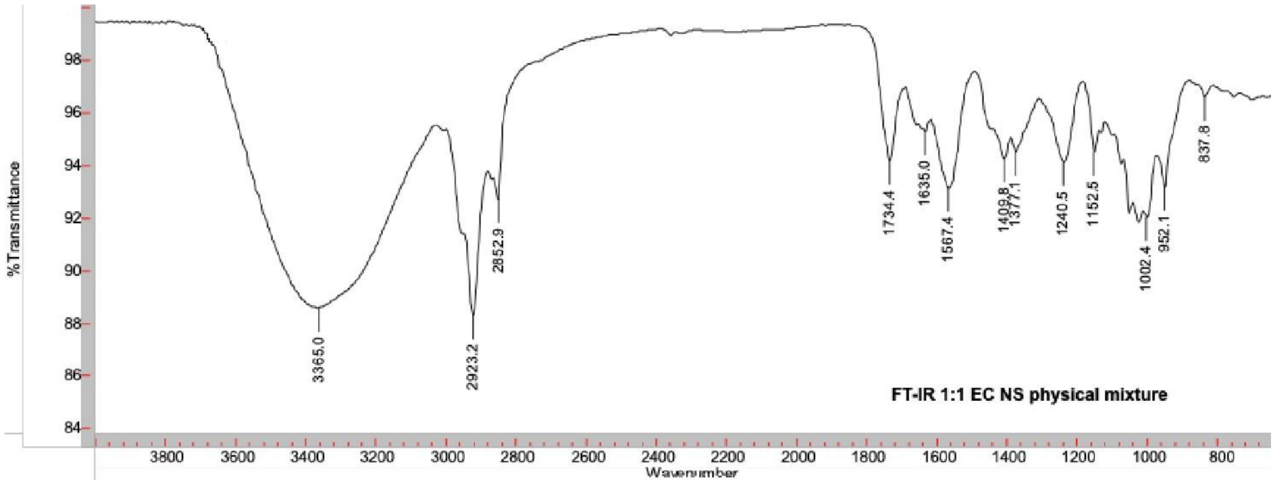

Figure S5. Superimposition of the ATR-FTIR spectra of euplotin C (red), the loaded EC-NS complex (green) and the binary 1:1 mixture of EC and NS (black).

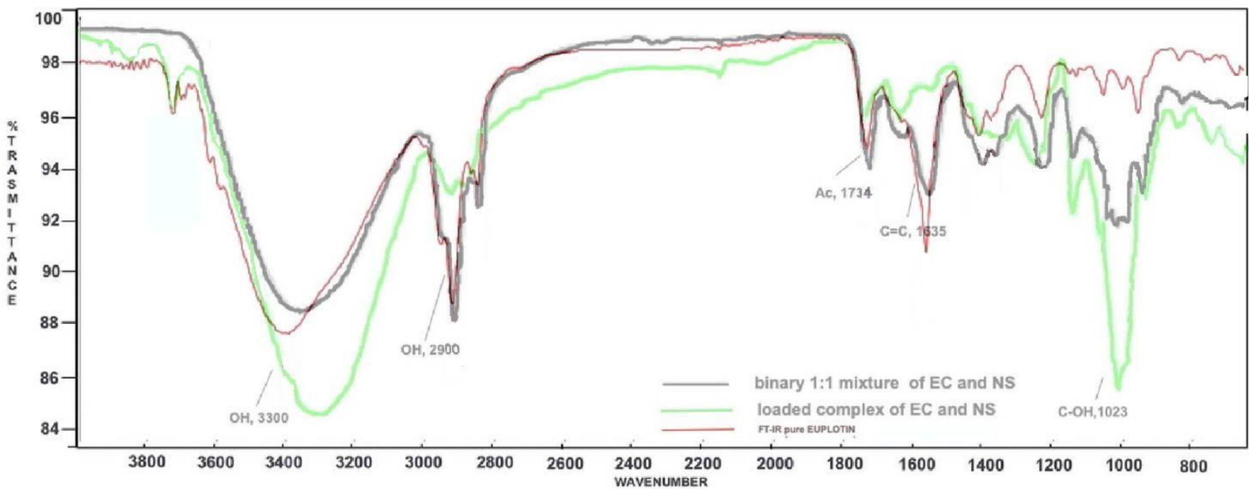

Figure S6. ^{1}H-NMR spectrum of β-cyclodextrin-based nanosponges, recorded in D_2O.

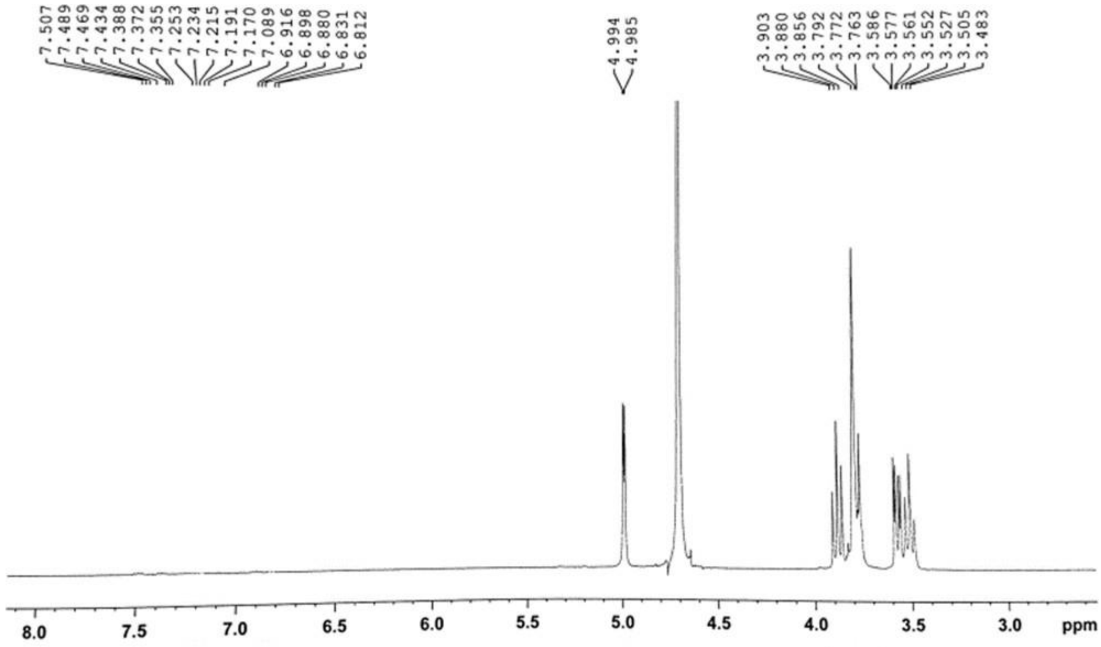

Figure S7. Graphical representation of the workflow putted in place to recover pure euplotin C from *E. crassus* SSt22 strain culture.

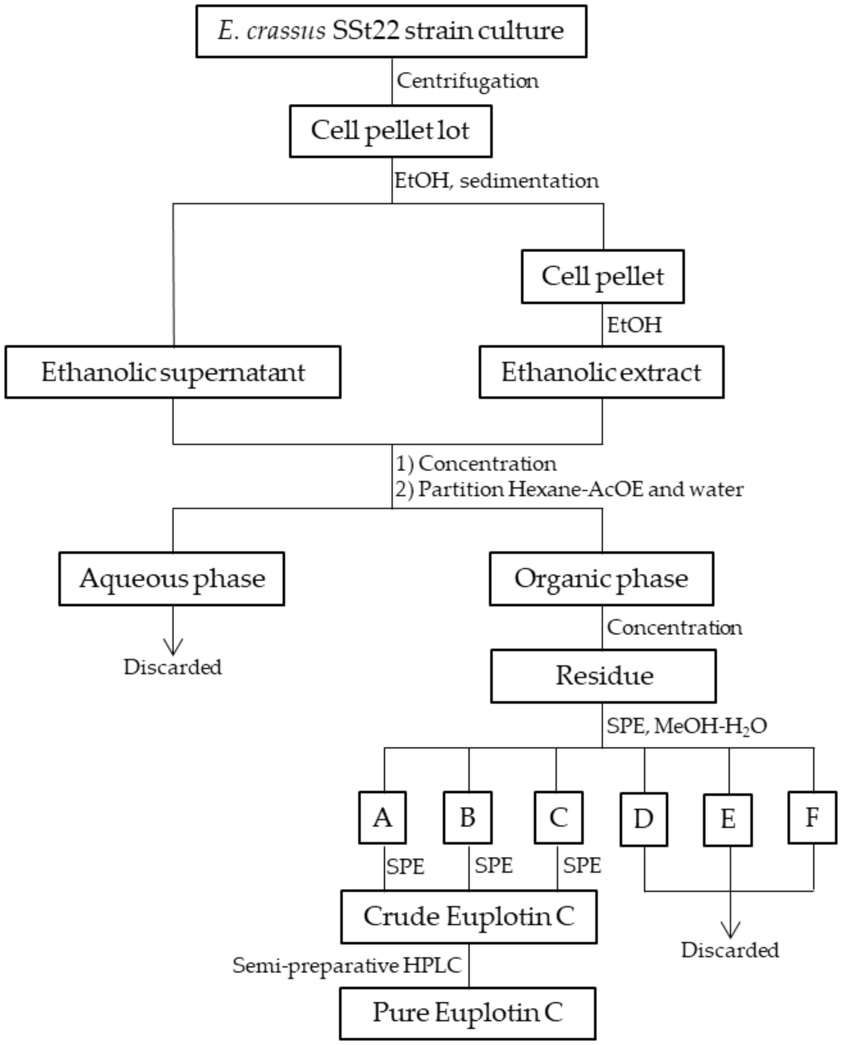

Figure S8. RP-C18-TLC Analysis of Samples A-F obtained by SPE C18 fractionation of *E. crassus* cell pellets extraction.

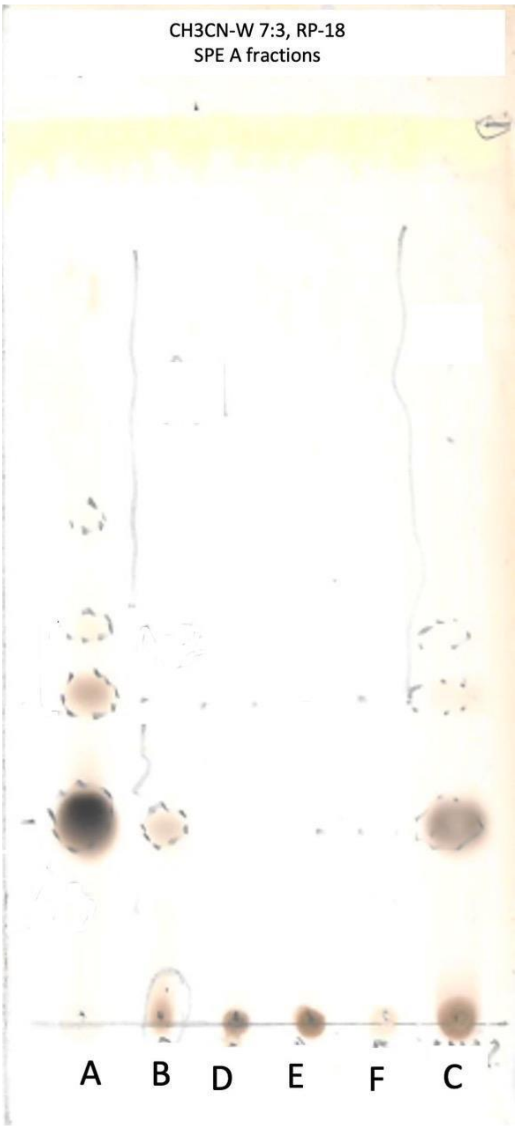

Figure S9. Purification of euplotin C fractions obtained by Solid Phase Extraction.

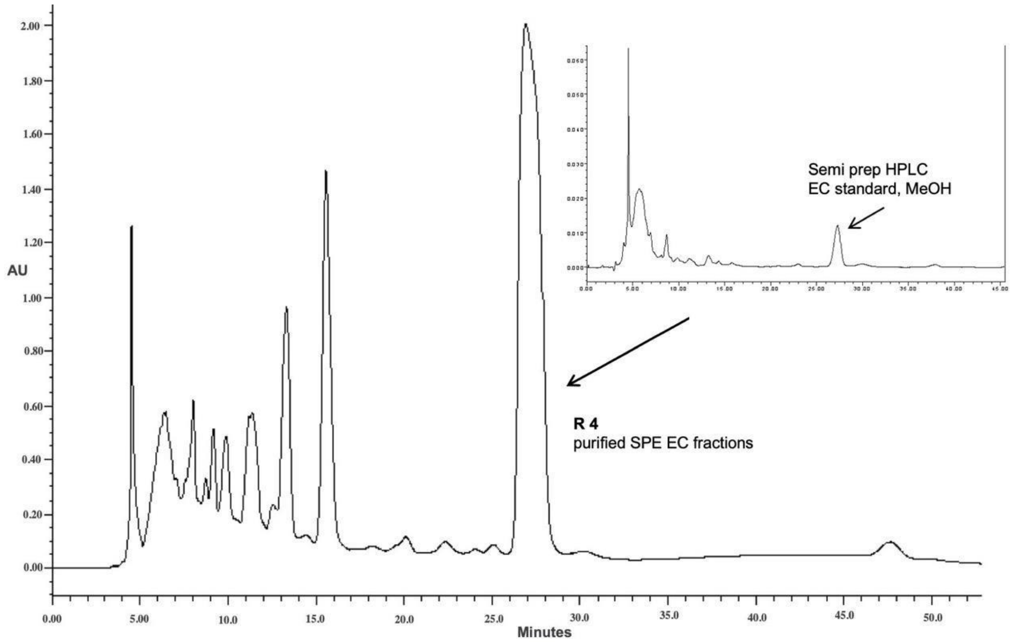

Figure S10. ^{1}H-NMR spectrum of euplotin C, recorded in $CDCl_3$-d_1.

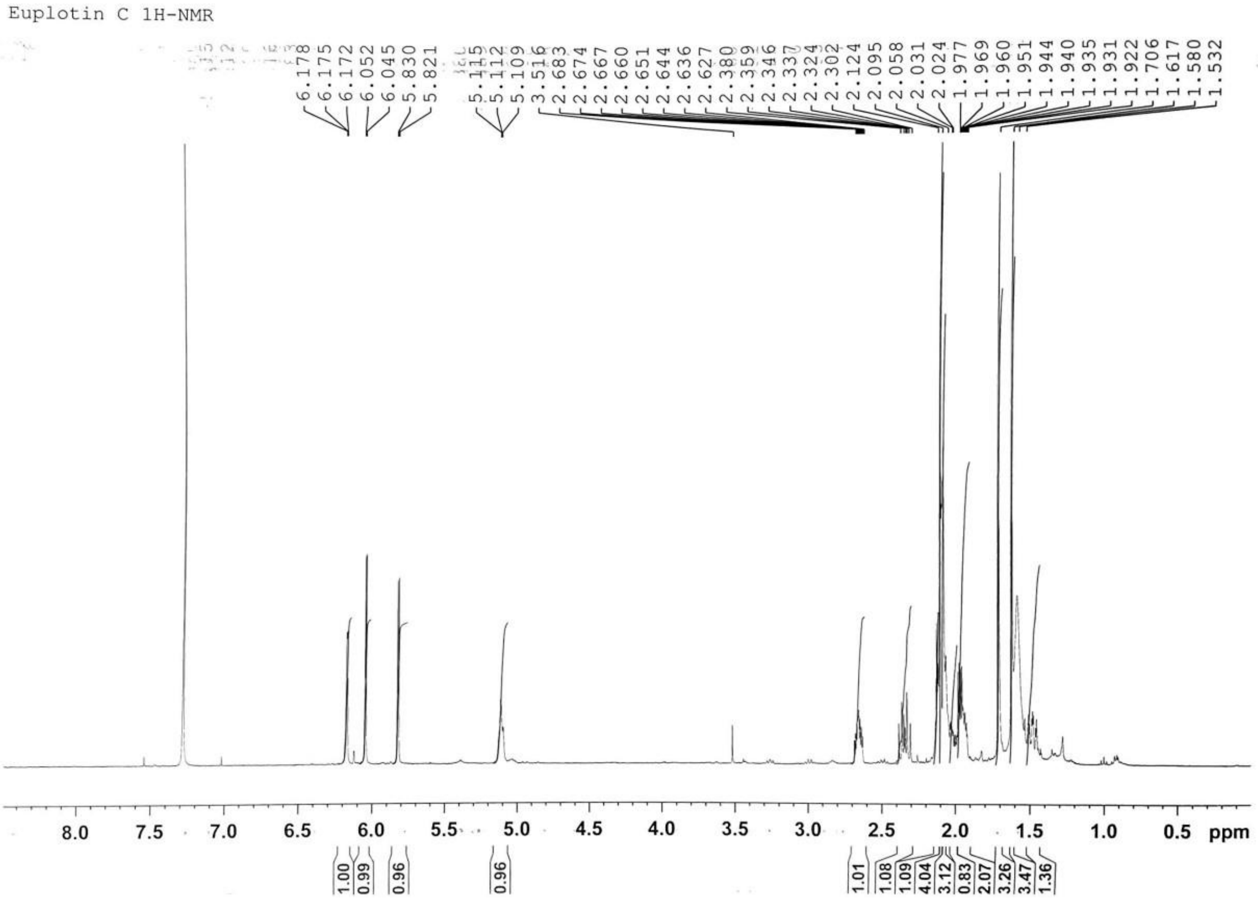

Figure S11. ^{13}C-NMR spectrum of euplotin C, recorded in $CDCl_3$-d_1.

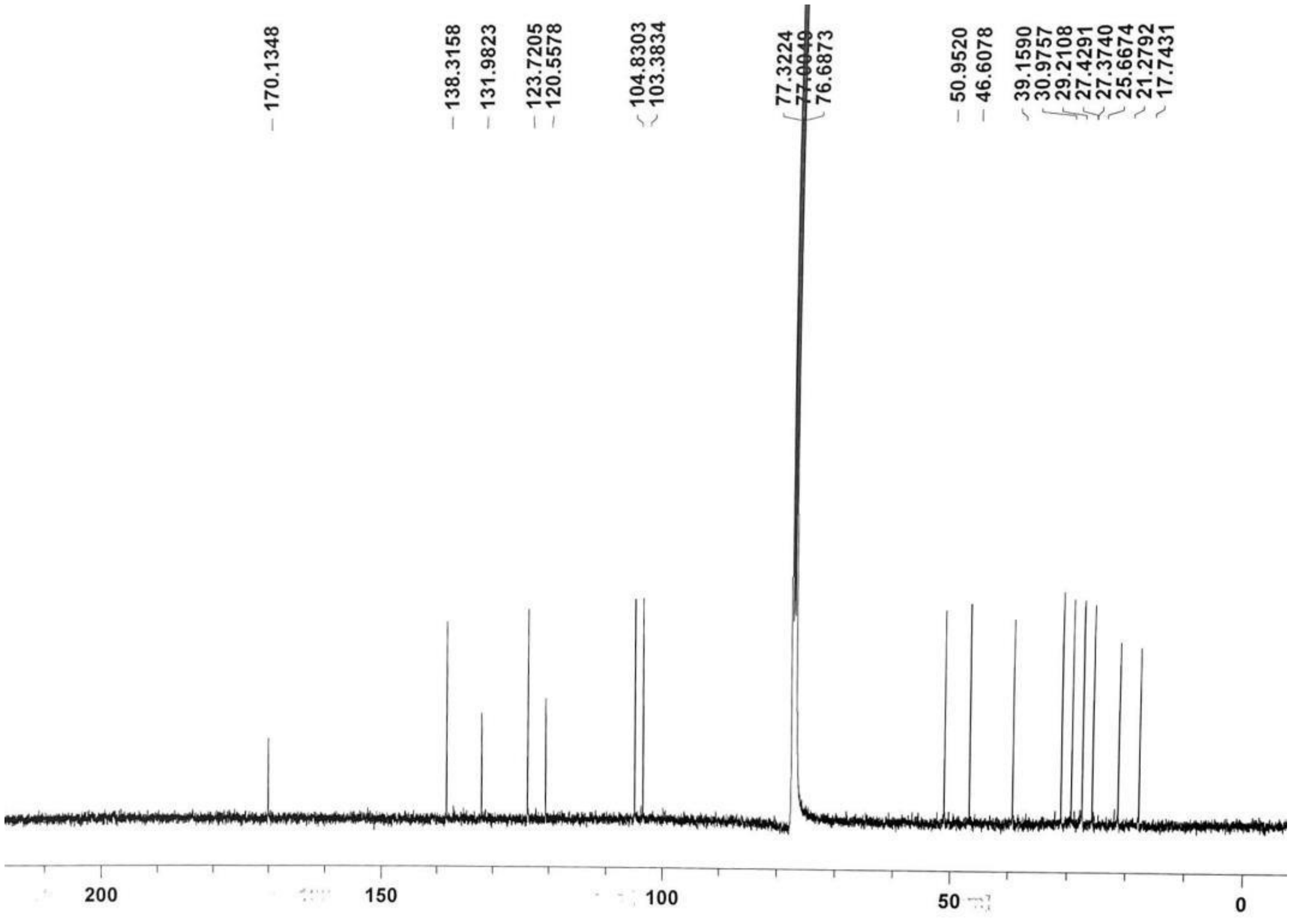

Figure S12. LC-PDA-ESI-MS analysis of euplotin C.

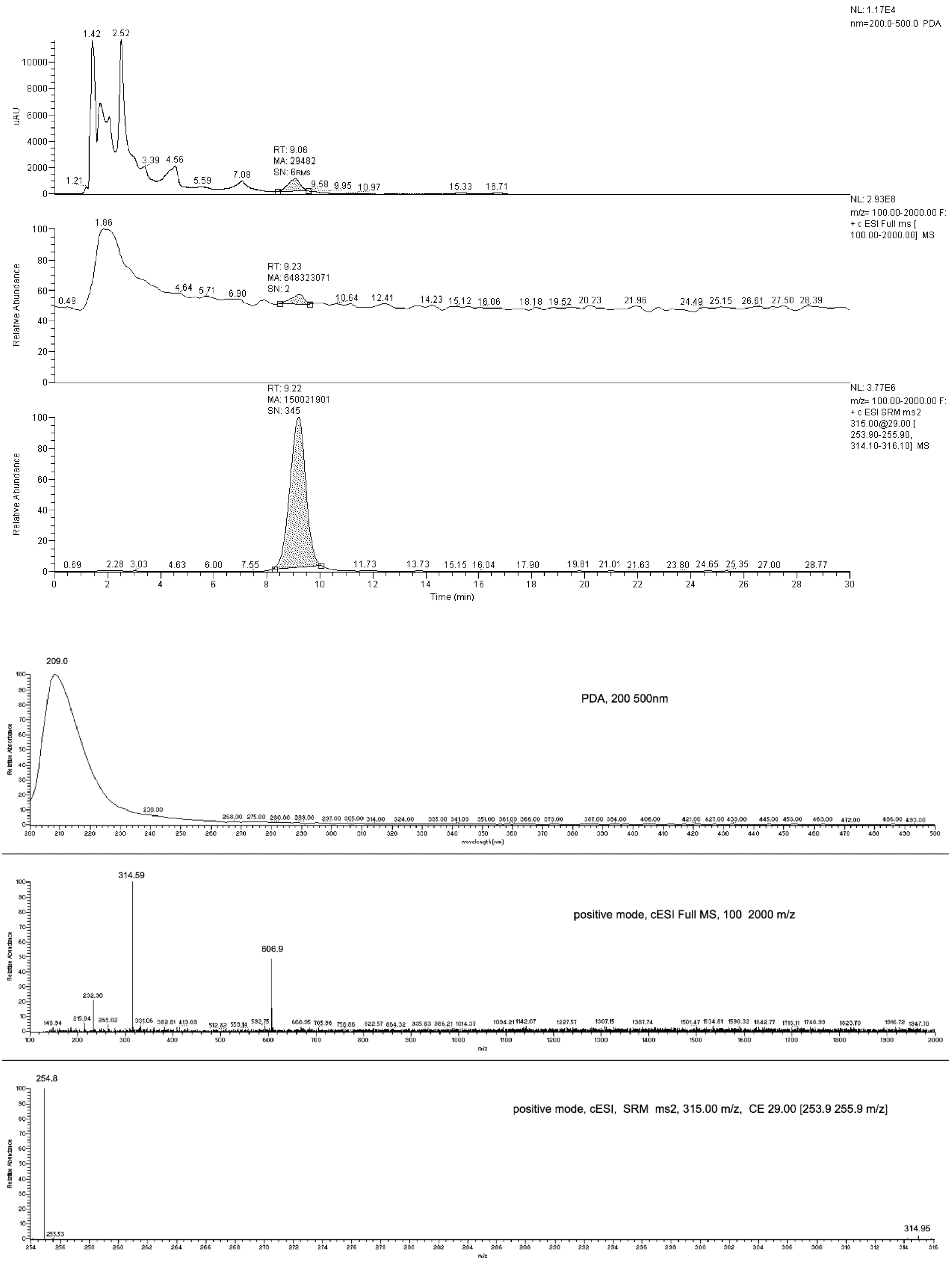

Table S1. Parent/adduct and daughter ions obtained by direct infusion ESI-MS analyses of unloaded β-cyclodextrin-based nanosponges (NS), monomeric β-cyclodextrin, and euplotin C extracted from loaded β-cyclodextrin-based nanosponges by means of either apolar (EC-NS1) or polar (EC-NS2) solvents.

Sample	Selected Parent/Adduct ions [M+H]+	Collision Energy	Daughter Ions
**NS**[a]	1347.8	43	1305.2 (25%)<br>1270.0<br>1026.5 (30%)<br>685.4 (100%)
	663.40	48	607.1 (70%)<br>551.1 (90%)<br>495.2 (30%)<br>463.2 (100%)
**NS**[b]	444.9 (100%)	38	428.8 (100%)<br>358.5 (100%)
	298.8 (98%)		
	370.9 (80%)	30	354.9 (100%)
	518.8 (50%)	45	502.8 (45%)<br>414.8 (20%)<br>354.9 (100%)
	663.5		607.1<br>551.1<br>495.1
**β-Cyclodextrin**	1157.5		
	663.4	35	607.2<br>551.1<br>494.8
	628.5	45	516.2
	549.3	40	495.0<br>383.2<br>377.1<br>355.0
	521.3	37	377.1<br>354.9<br>349.1<br>327.1
	493.3	36	348.9<br>327.0
**EC-NS1**[c]	1347.4 (40%)		
	977.3 (100%)	45	945.4 (25%)<br>918.5 (100%)<br>906.8 (45%)<br>778.6 (15%)<br>679.4 (50%)<br>426.9 (15%)
	607.2 (98%)		
	315.1 (65%)	30	254.8 (100%)
**EC-NS2**[d]	1215.4 (60%)	33	1139.3 (90%)<br>1053.2 (100%)<br>891.2 (45%)<br>833.2 (25%)<br>729.1 (38%)<br>671.1 (20%)
	1157.5 (80%)		995.3 (100%)<br>833.3 (90%)<br>671.2 (40%)<br>509.2 (15%)
	607.1 (50%)		

	561.3 (30%)	40	547.1 (70%)<br>533.1 (100%)<br>515.2 (35%)
	315.1 (100%)		

[a]Unloaded nanosponges treated with n-hexane/ethyl acetate 9/1 for 24 hours; [b]Unloaded nanosponges treated with MeOH for 24 hours; [c]euplotin C extracted from loaded nanosponges by treatment with n-hexane/ethyl acetate 9/1, for 24 hours; [d]euplotin C extracted from loaded nanosponges by treatment with MeOH, for 24 hours.
